# Case Report: Clinical and Genetic Characteristics of Pearson Syndrome in a Chinese Boy and 139 Patients

**DOI:** 10.3389/fgene.2022.802402

**Published:** 2022-05-23

**Authors:** Yanqin Ying, Yan Liang, Xiaoping Luo, Ming Wei

**Affiliations:** Department of Pediatrics, Tongji Hospital, Tongji Medical College, Huazhong University of Science and Technology, Wuhan, China

**Keywords:** Pearson’s syndrome, anemia, mitochondrial DNA deletion, clinical features, gene mutations

## Abstract

**Background:** Pearson’s syndrome (PS) is a rare multi-system disorder caused by mitochondrial DNA deletion. Most PS cases in the literature are individual reports, and there is a lack of systematic analysis of clinical features and gene mutations in large samples.

**Objective:** To report a case of PS and summarize the clinical features and genetic characteristics of PS by reviewing the literature.

**Methods:** We reported a case of PS in a boy with severe anemia and multi-system disorder. Genetic etiology was identified by mitochondrial DNA sequencing and whole-exon sequencing. Clinical features and gene mutations were summarized by literature review.

**Results:** The patient had major clinical manifestations with recurrent anemia and multiple organ failure after infection. Mitochondrial DNA sequencing revealed a *de novo* heteroplasmic deletion of 3.063 kb (nt 6,224–9,287) with 75% heteroplasmy in peripheral blood. A total of 139 PS cases were retrieved after a literature search. The most common initial symptom was refractory anemia requiring repeated blood transfusion (86.2%), digestive system symptoms (26.9%), and failure to thrive (15.4%). During the course of disease, the observed symptoms were bone marrow failure (100%), metabolic disorders (61.87%) and gastrointestinal symptoms (61.87%), failure to thrive (48.9%), renal disorders (42.45%), and pancreatic exocrine insufficiency (39.6%). The mean heteroplasmy of mitochondrial DNA mutation in peripheral blood in deaths (76.29 ± 11.86%, *n* = 29) was higher than that in survivals (59.92 ± 23.87%, *n* = 26, *p* < 0.01). Among the patients with the 4.977 kb deletion, the heteroplasmy in peripheral blood in deaths (79.64 ± 9.71%, *n* = 11) was higher than that in survivals (56.67 ± 27.65%, n = 9, *p* < 0.05).

**Conclusion:** PS can affect multiple systems, and mitochondrial DNA sequencing should be performed early. The heteroplasmy in peripheral blood is related to prognosis.

## Introduction

Pearson syndrome (PS, OMIM: 557,000), also known as Pearson marrow pancreas syndrome, was first reported by Pearson et al., in 1979 (Pearson et al., 1979). PS is a congenital multi-system disorder caused by mitochondrial DNA (mtDNA) mutation and characterized by severe anemia with neutropenia and (or) thrombocytopenia, ring sideroblasts in the bone marrow, pancreatic exocrine insufficiency, and hyperlactic acidemia (Rötig et al., 1990). Most patients tend to have symptoms in early infancy or even the neonatal period and die before the age of three. Some survivors develop Kearns–Sayre syndrome (KSS), which is characterized by ophthalmoplegia, ataxia, retinitis pigmentosa, conduction defects, and myopathy, while the hematological signs disappear (Giese et al., 2007; Tumino et al., 2011). PS is very rare mitochondriopathies, and mtDNA mutations are rather homogeneous since the same 4.977 kb deletion constitutes the most common lesion (Rötig et al., 1995)) and is generally sporadic. The true incidence of PS is still unknown. The current literature regarding PS mainly were individual reports and some reports summarized the clinical features (Manea et al., 2009; Pronman et al., 2019). There is a lack of large sample systematic analyses of the clinical features and the relationship between mtDNA mutations and clinical features. Here, we report one Chinese child with PS. In addition, we have also reviewed the literature to summarize the clinical features and gene mutation of PS.

## Case Report and Methods

### Case Data

**FIGURE 1:**
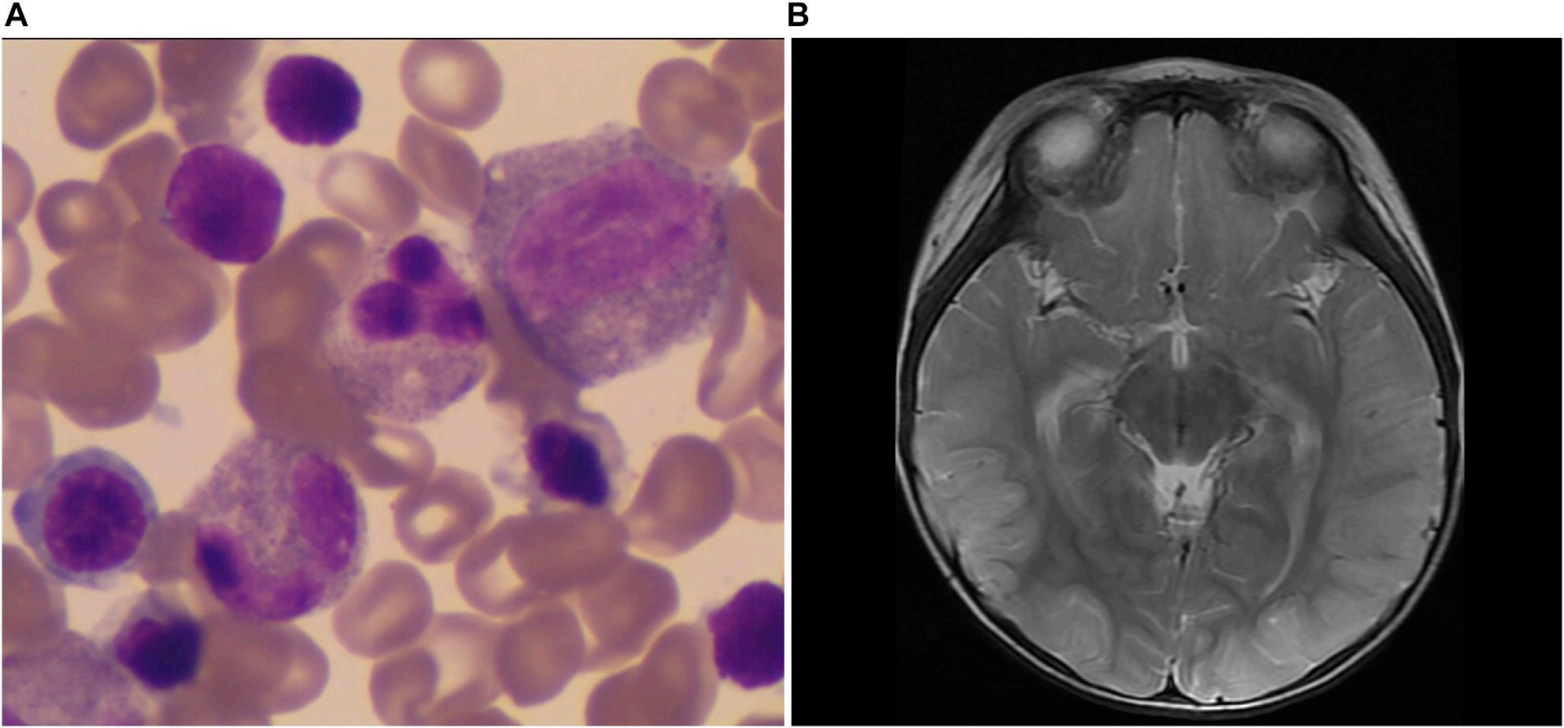
The patient’s main clinical characters. **(A)** Bone marrow aspirate showed markedly active myeloproliferation with impaired megakaryocyte maturation. **(B)** The brain DWI image. It showed linear hyperintensity in the left occipital lobe on diffusion-weighted imaging (DWI).

The patient was a male child with full-term natural birth and 2.5 kg of birth weight. His family history was unremarkable. At the age of 7 months, anemia was observed, and serum hemoglobin (Hb) levels was 80 g/L. No special treatment was given. At 10 months of age, he was admitted to the pediatric intensive care unit (PICU) because of fever and vomiting for 5 days. The blood examination revealed anemia (Hb 86 g/L), neutropenia (0.57×10^9^/L), and thrombocytopenia (71×10^9^/L). Serum ferritin levels was 121.7 ug/L (30–400 ug/l). Bone marrow aspirate showed markedly active myeloproliferation with impaired megakaryocyte maturation (Figure 1A). The patient had high levels of ALT (146 U/L) and AST (227 U/L). Pancreatic amylase level was 4 U/L (13–53 U/L), and lipase level was 226 U/L (13–60 U/L). Serum lactic acid level was 9.3 mmol/L (0.5–2.2 mmol/L), while blood glucose, serum ammonia were normal. No specific metabolites were detected by blood amino acids and tandem mass spectrometry of urine. The patient was discharged after 12 days of hospitalization upon symptoms relief. After the discharge, the growth and development of the child were normal, and he had frequent bowel movements.

At the age of 17 months, the patient was readmitted to the PICU for vomiting and a poor appetite. He had epileptic seizures and went into a coma. The blood examination revealed anemia and thrombocytopenia, while neutrophils were within the normal range. The patient had decreased amylase levels, hypoglycemia, metabolic acidosis, hyperlactic acidemia, increased ALT level, and increased ammonia level (673 umol/L). High-sensitivity cardiac troponin I and brain natriuretic peptide (BNP) increased to 160.3 pg/ml (<40 pg/ml) and 3,006 pg/ml, respectively. The second bone marrow aspiration also revealed sideroblast anemia. A brain diffusion-weighted imaging (DWI) scan (Figure 1B) showed linear hyperintensity in the left occipital lobe. Video electroencephalogram (VEEG) showed background diffuse δ slow waves, high-amplitude slow waves and sharp slow-wave rhythmic bursts in bilateral frontal and mid-frontal areas, while no sleep spindles were recorded. Whole-exon sequencing was performed, but no pathogenic gene mutations were detected. The patient was discharged after 18 days. At the time of discharge, his limbs muscle strength was decreased to grade III, and he was unable to walk. His stool frequency was increased.

One month later, the child was hospitalized for vomiting and anemia. Pupillary light reflexes were weak in both eyes. The hearing was normal. Pancreatic function exocrine function was reduced. Folic acid, vitamin B12, renal function, and tubular function proteins were all normal. The mitochondrial disease was suspected, and mitochondrial gene sequencing was performed.

The patient was administered multivitamins, L-carnitine, CoQ10, and other supportive therapies. After discharge, he was given repeated blood transfusions. His platelets and neutrophils were within the normal range, and he still had metabolic acidosis, hyperlactic academia, and elevated liver enzymes. At 25 months of age, his muscle strength was increased, and his pupillary light reflex became normal. There was no significant lag in growth and mental development. At 31 months of age, the patient died from a severe infection.

### Whole-Exon Sequencing and Whole Mitochondrial Genome Sequencing Analysis

Four milliliter of venous blood was drawn from the child and the parents. Both nuclear genome and mitochondrial genome sequencing in peripheral blood proceeded as previous publication (Liang et al., 2020). In brief, The fragmented DNA library was captured using GenCap of 23,000 genes and Mitochondrial DNA Capture Kits. Sequencing was carried out using the Illumina HiSeq X ten platform for paired-reading of 150 bp. After sequencing, the raw data were saved in a FASTQ format. Both Illumina sequencing adapters and low-quality reads were filtered using the cutadaptor software (http://code.google.com/p/cutadapt/). The clean reads of nuclear genome were aligned to human reference genome (hg19, UCSC) using the sentieon software (https://www.sentieon.com/). The clean reads of mitochondrial genome were aligned to each human reference (hg38, UCSC) using the sentieon software (https://www.sentieon.com/). Duplicated reads were removed, and only unique mapping reads were used for the next variation detection. Both SNVs and Indels of the nuclear genome and mitochondrial genome were detected using the HaplotypeCaller module of the GATK software (https://software.broadinstitute.org/gatk/). In addition, the relative copy numbers were calculated using the tools of fix, segment, and call of the CNVkit software (https://cnvkit.readthedocs.io/en/stable/).

### Ethical Approval

The study was approved by the Ethics Committee of Tongji Hospital, Tongji Medical College of Huazhong University of Science and Technology.

### Literature Search

**FIGURE 2:**
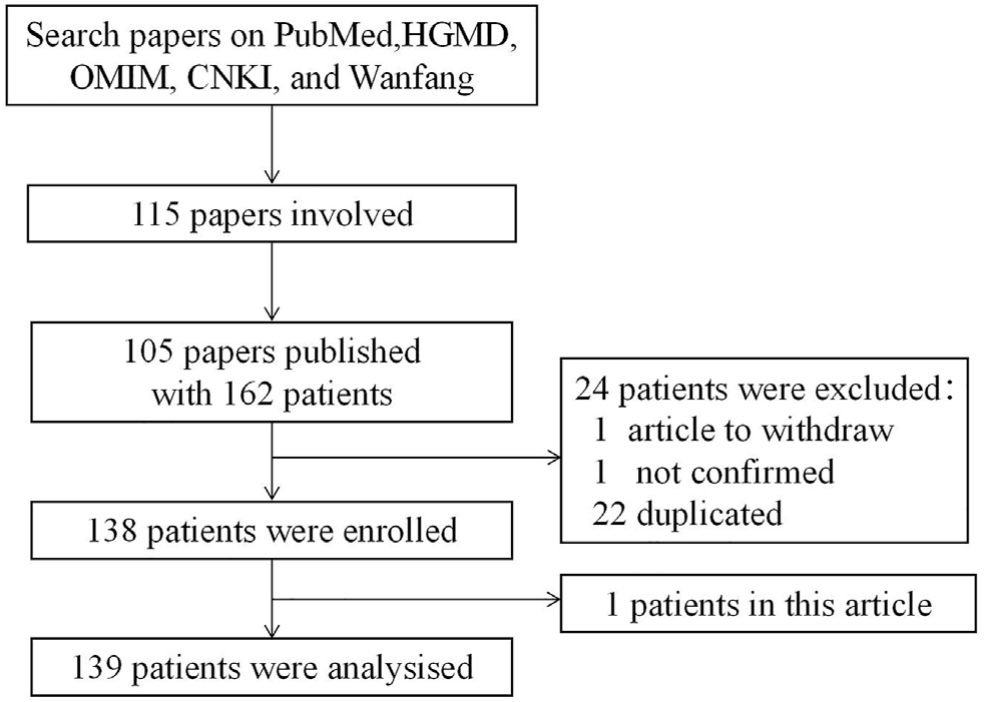
Case-screening flowchart.

Based on PubMed, Human Gene Mutation Database (HGMD), Online Mendelian Inheritance in Man (OMIM), China National Knowledge Infrastructure (CNKI), and Wanfang Data searches using keywords “Pearson marrow pancreas syndrome”, “mtDNA deletion syndrome” and “Pearson syndrome”, we identified and compiled 105 papers containing PS case reports published before March 2021. After removing duplicates, 139 PS cases with a complete medical history and gene mutation data were obtained (Figure 2). The onset age was defined as the time the first symptom was recorded in study. The age of diagnosis referred to the age that the genetic mutation was confirmed or the PS was diagnosed in the study.

All the patients were divided into survivals and deaths groups according to the status at the end of the follow-up period.

### Statistical Analysis

Descriptive statistical analysis was used for clinical features. The mtDNA heteroplasmy was expressed as a percentage. The heteroplasmy from peripheral blood in the deaths and survivals groups were expressed as mean ± SD. The comparison of gene heteroplasmy between the two groups was performed using two-sided *t-*tests, and *p* < 0.05 was considered statistically significant.

## Research Results

### Genetic Test Results

**FIGURE 3:**
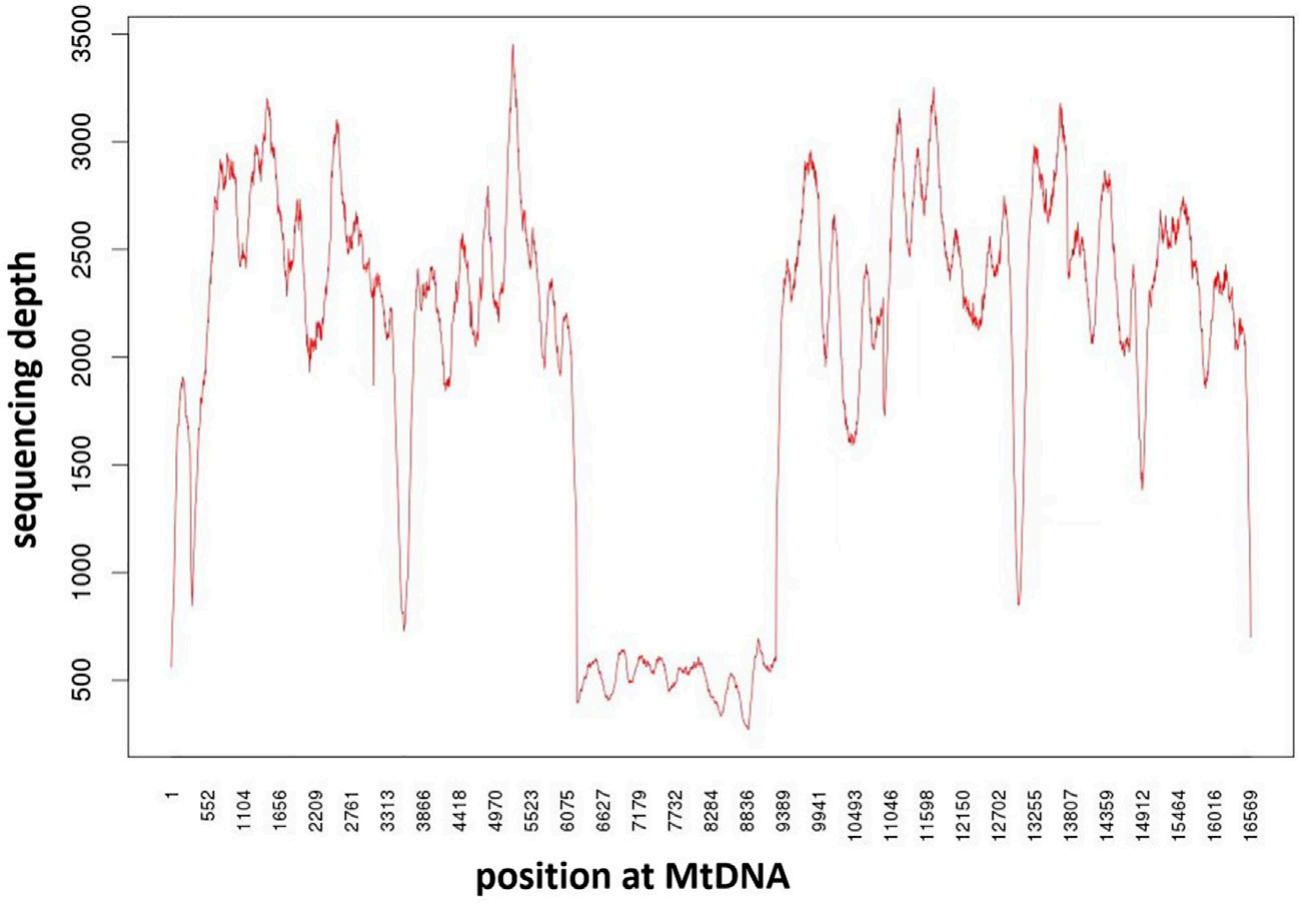
Mitochondrial DNA mutation in the patients. Analysis of mitochondrial DNA mutations in the child’s blood showed a heteroplasmic deletion of mitochondrial DNA 3.063 kb (nt 6,224–9,287).

No pathogenic gene mutations were detected by whole-exon sequencing. The mtDNA sequencing indicated that the patient had a 3.063 kb deletion in the area of chrM: 6,224–9,287 (Figure 3), including part of MT-CO1, all MT-TS1, MT-TD, MT-CO2, MT-TK, MT-ATP8, MT-ATP6, MT-CO3, and the heteroplasmy was 75% in peripheral blood. The mother didn’t carry the same mutation. It was a ***de novo*** mutation since this mutation had not been reported and published at mitobreak (http://mitobreak.portugene.com/cgi-bin/Mitobreak_home.cgi/).

### General Characteristics of Pearson Syndrome

Among the 139 PS cases, a total of 131 cases had clear gender records, which included 60 females and 71 males (Supplementary data S1).

**FIGURE 4:**
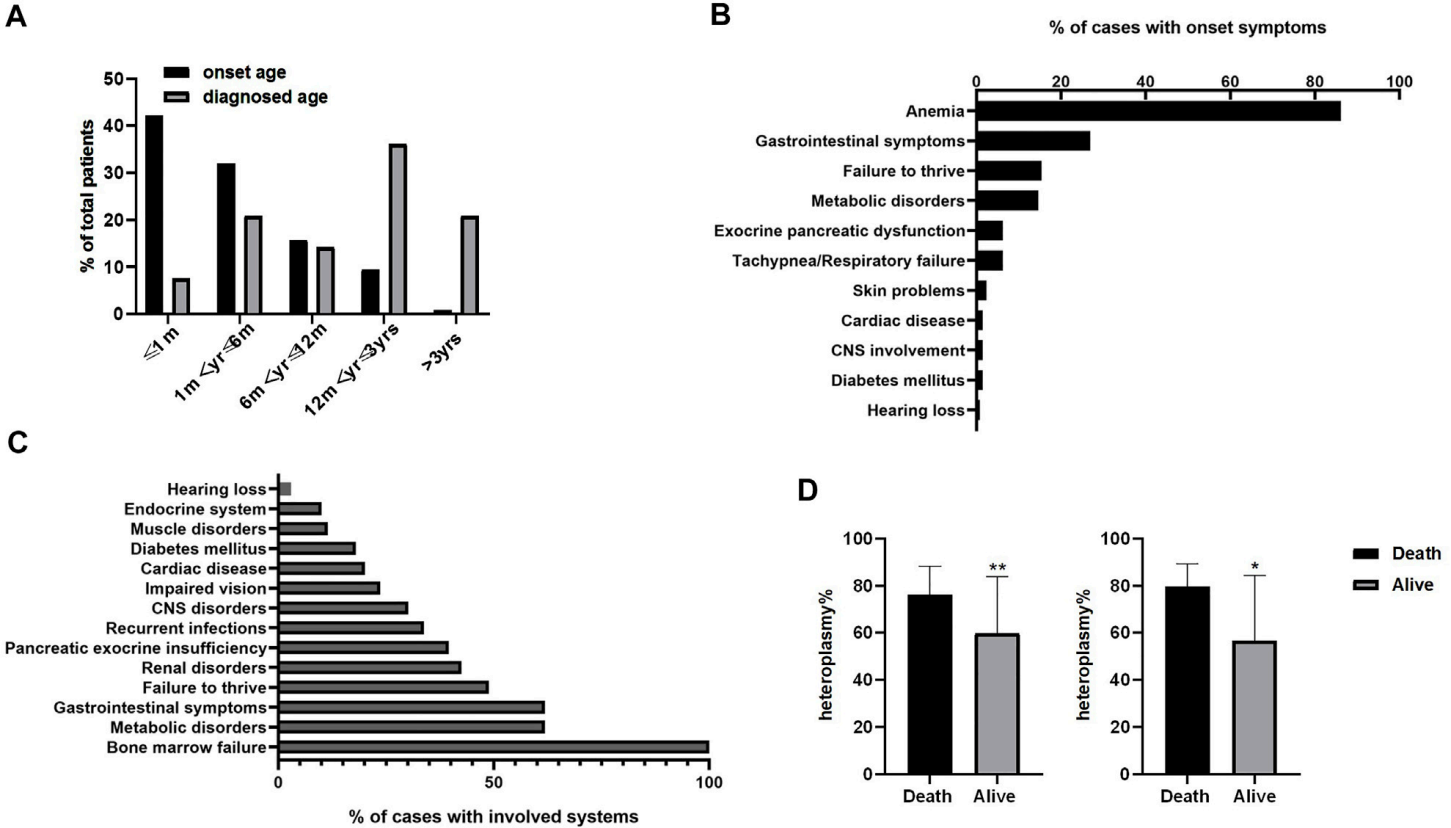
Disease characteristics of 139 patients. **(A)** Age of onset and diagnosis time from the onset in Pearson syndrome patients. **(B)** The percentage of initial symptoms observed in patients. **(C)** The percentage of symptoms observed in patients during the course of the disease. ^a^ refers to diarrhea and vomiting;^b^ refers to hypothyroidism, growth hormone deficiency, and adrenal insufficiency. **(D)** Comparison of mitochondrial mutation heteroplasmy in peripheral blood between the death group and the survival group. The mean heteroplasmy of mitochondrial DNA mutation in peripheral blood was statistically higher in the deaths (76.29 ± 11.86%, *n* = 29) than that in survivals (59.92 ± 23.87%, *n* = 26; *t* = 3.272, *p* < 0.01. left). For patients with 4.977 kb deletion (right), the mean heteroplasmy of mitochondrial DNA mutation genes in peripheral blood was statistically higher in the deaths (79.64 ± 9.71%,*n* = 11) than that in survivals (56.67 ± 27.65%, *n* = 9) (*t* = 2.58, *p* < 0.05). Error bar represents the SD of heteroplasmy from peripheral blood in the deaths and survivals groups. Supplementary data S1 The data of patients summarized from the literature review. Supplementary data S2 Correlation between heteroplasmy of mitochondrial DNA deletion in peripheral blood and the age of onset. Supplementary data S3 Correlation between heteroplasmy of 4.977 kb deletion in peripheral blood and the age of onset.

The onset age was mostly within 1 month after birth (54/128, 42.2%), followed by 1 month–6 months (41/128, 32.0%), Figure 4A. While only 8/105 (7.6%) patients were diagnosed within 1 month of age. Most patients were diagnosed after 1 year of age (60/105, 57.2%).

From onset to diagnosis, the most common duration was 1–3 years (27/103, 26.2%), followed by less than 1 month (23/103, 22.3%). Out of 139 patients, 130 had documented outcomes. Among them, total of 69 cases (52.3%) died, and 17 cases (24.6%) died within 1 year of age, 32 cases (46.4%) died within 1–3 years, and 20 cases died after 3 years of age (29.0%). Among the surviving cases, 2 cases developed to KSS.

Pregnancy and birth history were recorded in 67 cases, which included 4 cases (6.0%) with preterm delivery, 7 cases (10.4%) with intrauterine growth restriction, 5 cases (7.5%) with intrauterine distress, 2 cases (3.0%) with meconium-stained amniotic fluid, and 2 cases (3.0%) with oligohydramnios during pregnancy. Only 57 cases with the record of family history were reported. Among them, four cases had related family history including 1 case with a maternal diagnosis of progressive external ophthalmoplegia (PEO), 1 case with the death of the first three fetuses in the family, 1 case with tremors in the mother and grandmother, 1 case with the family history of PS in a sibling. One case had an unrelated family history with sudden infant death syndrome in paternal uncle.

### Initial Symptoms

There were 130 cases with clearly recorded initial symptoms (Figure 4B). The most common initial symptom was refractory anemia requiring repeated blood transfusions (112 cases, 86.2%). Among the patients with anemia, there were 27 cases (20.8%) of anemia with neutropenia or thrombocytopenia. None of the patients had initial symptoms as separated neutropenia or thrombocytopenia. Anemia was the only initial symptom in 58 cases (44.6%). In addition, there were 35 cases (26.9%) with gastrointestinal symptoms, including vomiting, diarrhea, and feeding difficulties, 20 cases (15.4%) with failure to thrive, 19 cases (14.6%) with metabolic disorders, including metabolic acidosis, hyperlactic acidemia, hypoglycemia, and electrolyte disorders, and 8 cases (6.2%) with pancreatic exocrine insufficiency.

### Symptoms Observed During the Course of the Disease

Multiple symptoms were observed in 139 cases during the course of the disease (Figure 4C). Bone marrow failure was reported in all 139 cases (100%), presenting as refractory anemia with or without neutropenia and (or) thrombocytopenia. Both metabolic disorders and gastrointestinal symptoms occurred in 86 cases (61.9%), failure to thrive in 68 cases (48.9%), renal disorders in 59 cases (42.5%), pancreatic exocrine insufficiency in 55 cases (39.6%), recurrent infections in 47 cases (33.8%), impaired vision with ocular and limb muscles disability in 33 patients (23.7%), central nervous system affected in 42 patients (30.2%), cardiac disease in 28 cases (20.1%), and diabetes mellitus in 25 cases (18.0%).

### Mitochondrial DNA Mutation

Among the 139 PS cases, mitochondrial DNA mutation was documented in 135 cases, all of which were heteroplasmy mutations, including 8 cases with duplication in addition to heteroplasmic deletion. There were 30 cases (22.2%) of 4.977 kb deletion and 105 cases (77.8%) of other mitochondrial DNA mutations. Mitochondrial gene deletion sites were between nt.6074 and nt.16082, and the genes involved included MT-CO1, MT-TS1, MT-TD, MT-CO2, MT-TK, MT-ATP8, MT-ATP6, MT-CO3, MT-TG, MT-ND3, MT-TR, MT-ND4L, MT-ND4, MT-TH, MT-TS2, MT-TL2, MT-ND5, MT-ND6, MT-TE, MT-CYB, MT-TT, and MT-TP.

### Mitochondrial DNA Deletion Heteroplasmy

Heteroplasmy in peripheral blood and/or bone marrow was detected in 85 cases, ranging from 20 to 100%. There was no significant correlation between heteroplasmy of mtDNA deletion in peripheral blood and age of onset, and heteroplasmy of 4.977 kb deletion in peripheral blood and age of onset (Supplementary data S2 and Supplementary data S3). The mean heteroplasmy of mtDNA mutation in peripheral blood was statistically higher in the deaths (76.29 ± 11.86%, *n* = 29) than that in survivals (59.92 ± 23.87%, *n* = 26; *t* = 3.272, *p* < 0.01), Figure 4D left. For patients with 4.977 kb deletion, the mean heteroplasmy of mtDNA mutation genes in peripheral blood was statistically higher in the deaths (79.64 ± 9.71%, *n* = 11) than that in survivals (56.67 ± 27.65%, *n* = 9) (*t* = 2.58, *p* < 0.05), Figure 4D right.

## Discussion

Pearson’s syndrome is a maternally inherited disease caused by the heteroplasmic deletion of mitochondrial DNA. The mutation leads to mitochondrial respiratory chain dysfunction and insufficient cellular energy supply (Smith et al., 1995; Williams et al., 2012). Most cases are sporadic, and those with a clear family history are rare. In this case, the patient had no obvious family history, and the mother had no symptoms of mitochondrial pathology. Among the 139 cases reported in the literature, only 57 cases had family history provided. Five cases had a clear family history. Due to genetic heteroplasmy, clinical manifestations were variable among siblings (Köklü et al., 2010).

PS has an early onset and can even occur in the uterus with anemic fetal hydrops and cardiomegaly (Manea et al., 2009). The onset of illness within 1 month after birth accounted for 42.2% of cases, and the age of diagnosis was mostly after 1 year of age, indicating that most patients got delayed diagnoses. Severe refractory anemia is a common initial presentation. Some children develop neutropenia and/or thrombocytopenia during the course of the disease. Pancytopenia was reported as an initial symptom in some cases (Falcon and Howard, 2017; Tadiotto et al., 2018). In this case, the patient’s initial presentation was an episode of moderate anemia. After 10 months, the patient required repeated blood transfusions. In the literature review, refractory anemia as the initial symptom accounted for 44.6% of cases. It was closed to 36.4% from patients with neonatal onset (Manea et al., 2009). Refractory anemia can easily be misdiagnosed as Diamond-Blackfan anemia (DBA) or other hematological disorders (Gagne et al., 2014). Gastrointestinal symptoms, including diarrhea, vomiting, and feeding difficulties were among the initial symptoms for 26.9% of cases. Only a small number of patients had gastrointestinal symptoms as the only initial symptoms (Niaudet et al., 1994). Only 6.2% of cases had initial symptoms of pancreatic exocrine insufficiency. There were 2 cases (1.5%) with pancreatic endocrine dysfunction, resulting in hyperglycemia and diabetes mellitus. Metabolic disorders, including hyperlactic academia and metabolic acidosis as initial symptoms, occurred in 19 patients (14.6%). It was 9% from patients with neonatal onset (Manea et al., 2009).

Multiple symptoms were observed as the disease progresses. In this case, several disorders were happened, including pancreatic exocrine function insufficiency, gastrointestinal disorders, metabolic imbalance, vision disorder, and central nervous disorders, while growth and renal function were spared. Based on the literature review, hematological symptoms was present in all patients, presented as anemia with or without neutropenia and/or thrombocytopenia. It was reported that the percentage of anemia, neutropenia and thrombocytopenia was 98%, 67% and 73%, respectively (Pronman et al., 2009). Bone marrow aspiration reveals ring sideroblasts or vacuolization of bone marrow precursor cells. In some patients, early bone marrow biopsy often fails to show these characteristic changes, and multiple biopsies are required (Pearson et al., 1979; Superti-Furga et al., 1993; Mkaouar-Rebai et al., 2013). Besides, the diagnosis may be missed by bone marrow examiners with no relevant experience. For our patient, in two bone marrow aspirations, the presence of active myeloproliferation and visible sideroblasts were typical factors, while the diagnosis was delayed. In most cases, gastrointestinal symptoms, such as diarrhea, elevated liver enzymes, cholestasis, enlarged liver, and liver failure, are responsible for mortality (Rötig et al., 1990; Rötig et al., 1995). Recurrent infection was happened in 42.5% of patients, it was not reported by other publications. Many patients died of multiple organ failure caused by infection (Crippa et al., 2015). Renal disorders (42.5%)was mainly manifested as Fanconi syndrome, characterized by aminoaciduria, proteinuria, diabetes mellitus, and electrolyte disturbance, with increased renal volume on Ultrasonography (McShane et al., 1991; Lichter-Konecki et al., 1993; Santorelli et al., 1996; Crippa et al., 2015). The percentage of renal disorder, pancreatic exocrine insufficiency and cardiac disease were similar to those reported by literature (Pronman et al., 2009). Mitochondrial inheritance is characterized by heteroplasmy and threshold effect, leading to diverse clinical phenotypes. The possibility of PS should be considered in children with anemia associated with other systemic changes.

PS is mainly caused by the heteroplasmic deletion of mitochondrial DNA. In this case, there was a *de novo* heteroplasmic deletion of 3.063 kb (nt 6,224–9,287), including part of MT-CO1, all MT-TS1, MT-TD, MT-CO2, MT-TK, MT-ATP8, MT-ATP6, MT-CO3. The heteroplasmy was 75% in peripheral blood. The common gene mutation is a heteroplasmic deletion of 4.977 kb (nt 8,489–13,447) which is involved in mitochondrial energy metabolism and electron transfer (Rötig et al., 1990; Rötig et al., 1995). This deletion mutation was found in 22.2% of the cases in the literature, while other uncommon deletions accounted for 77.8%. It was suggestive of diversity in gene mutation of PS. The different sizes of the deleted segments and the different effects on mitochondrial respiratory chain function may be one of the reasons for the diverse clinical presentation. The clinical manifestations were also affected by the heteroplasmy of mitochondrial mutations (McShane et al., 1991). Limited autopsy cases have shown heteroplasmy in mitochondrial deletion in other tissues that are different from those in bone marrow or peripheral blood (Superti-Furga et al., 1993; Jacobs et al., 2004). The heteroplasmy of mitochondrial deletions in peripheral blood is not significantly correlated with the age of onset and may be related to the different sizes of mitochondrial deletion fragments and different mitochondrial genes. There was no correlation between genetic heteroplasmy of 4.977 kp deletion in peripheral blood and age, indicating that there might be some other factors involved, such as heteroplasmy of mitochondrial deletion in other tissues and organs. Since the data of these PS cases were collected from the literature, there was no further analysis of the correlation between 4.977 kb gene deletion and severity of anemia. In addition, due to the unbalanced distribution of mitochondrial mutations in cell division, whether the heteroplasmy at a certain time can represent the initial level needs to be further studied. Our results showed that regardless of mitochondrial fragment size, the percentage of mitochondrial heteroplasmy in peripheral blood of the death was significantly higher than that in survivals. Since most children died from septic shock and multiple organ system failures, the heteroplasmy of other tissues may also be a factor affecting mortality. The specific threshold of mitochondrial DNA deletion in PS was still not clear. The results of blood heteroplasmy have shown that the minimum mitochondrial heteroplasmy is 15% (Rötig, A. et al., 1995).

There was a lack of effective treatment with a poor prognosis and high mortality. Children with anemia require repeated blood transfusions. Most patients died due to the metabolic crisis and multi-system failure after infection. The mortality was 52.3% among the reported cases, and the majority of deaths were among infants and young children under 3 years of age. The leading causes of death were septic shock, multiple organ failure, and leukemia. Anemia was corrected in survivors without repeated blood transfusions, and adult cases developed KSS. If the symptoms of bone marrow failure are corrected early, patients can survive until 3 years of age, improving the prognosis. In this case, the patient needed repeated blood transfusions and still developed metabolic acidosis, hyperlactic academia, and elevated liver enzymes despite normal levels of platelets and neutrophils. The patient died at 31 months of age due to a severe infection.

In conclusion, this case report enriches the mitochondrial gene mutation spectrum and clinical features of PS. The literature review found that in addition to hematological and pancreatic dysfunction, PS also has a high incidence of gastrointestinal and renal tubular diseases. In clinical practice, if refractory anemia is accompanied by other systemic manifestations, the possibility of PS should be highly suspected.

## Data Availability

The datasets for this article are not publicly available due to concerns regarding participant/patient anonymity. Requests to access the datasets should be directed to the corresponding author.
