# Supplementary figures and images for: Case Report: Clinical and Genetic Characteristics of Pearson Syndrome in a Chinese Boy and 139 Patients
Source: Front Genet. 2022 May 23;13:802402. doi: 10.3389/fgene.2022.802402 (PMC9168460; doi:10.3389/fgene.2022.802402)

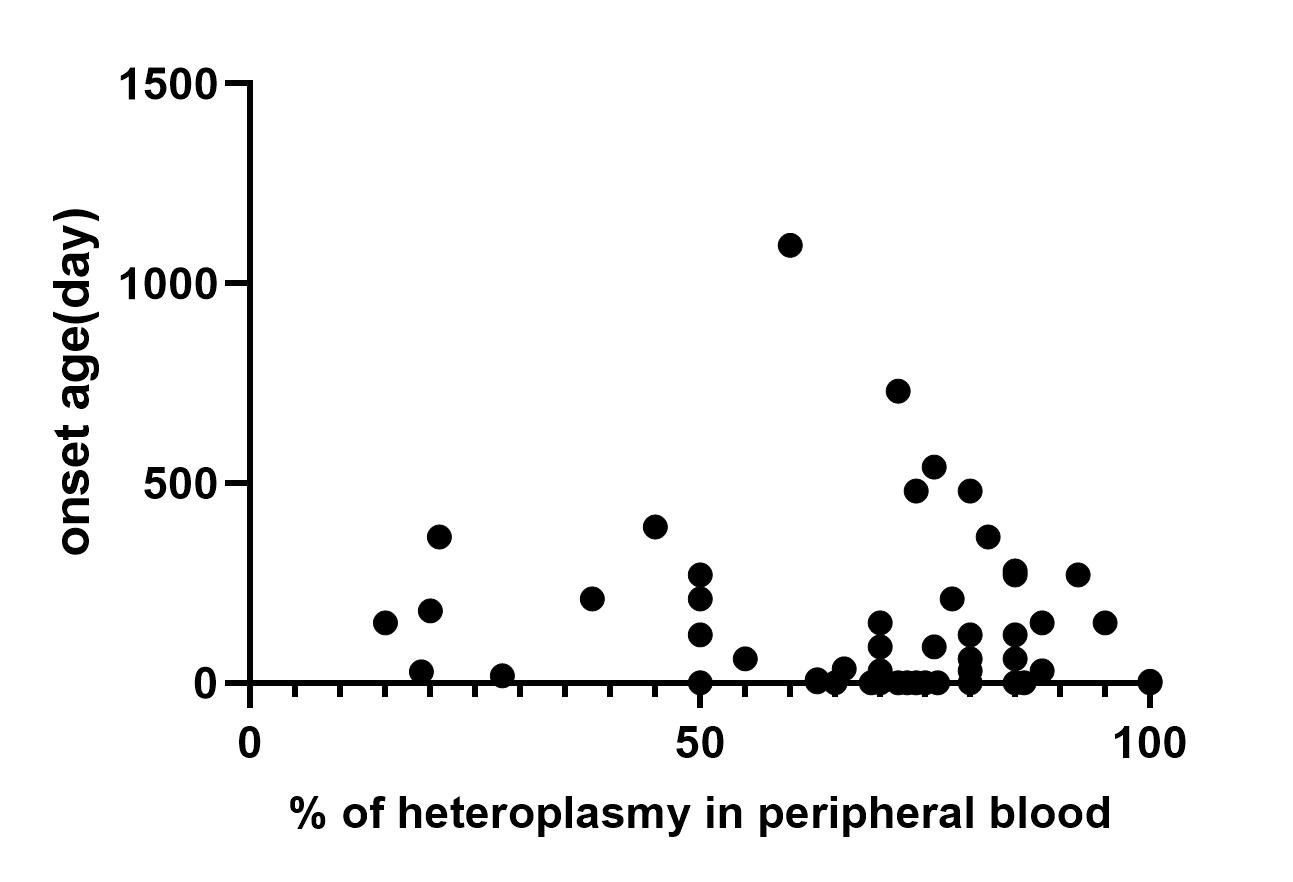

Supplement: Supplementary file 2 [file Image1.JPEG]

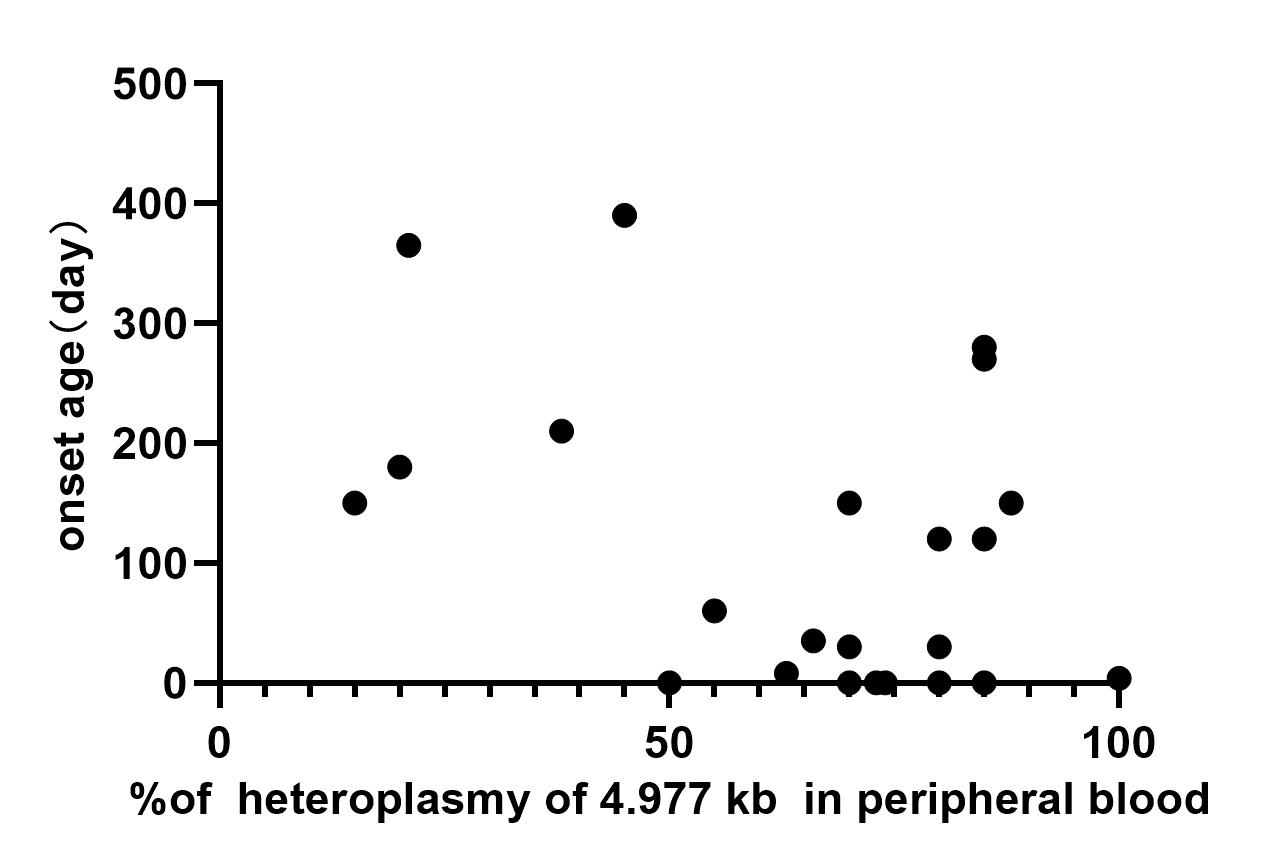

Supplement: Supplementary file 3 [file Image2.JPEG]
